# Automatic Breast Tumor Diagnosis in MRI Based on a Hybrid CNN and Feature-Based Method Using Improved Deer Hunting Optimization Algorithm

**DOI:** 10.1155/2021/5396327

**Published:** 2021-07-16

**Authors:** Weitao Ha, Zahra Vahedi

**Affiliations:** ^1^College of Computer, Weinan Normal University, Weinan, Shaanxi, China; ^2^Department of Electrical and Computer Engineering, Science and Research Branch, Islamic Azad University, Tehran, Iran

## Abstract

Breast cancer is an unusual mass of the breast texture. It begins with an abnormal change in cell structure. This disease may increase uncontrollably and affects neighboring textures. Early diagnosis of this cancer (abnormal cell changes) can help definitively treat it. Also, prevention of this cancer can help to decrease the high cost of medical caring for breast cancer patients. In recent years, the computer-aided technique is an important active field for automatic cancer detection. In this study, an automatic breast tumor diagnosis system is introduced. An improved Deer Hunting Optimization Algorithm (DHOA) is used as the optimization algorithm. The presented method utilized a hybrid feature-based technique and a new optimized convolutional neural network (CNN). Simulations are applied to the DCE-MRI dataset based on some performance indexes. The novel contribution of this paper is to apply the preprocessing stage to simplifying the classification. Besides, we used a new metaheuristic algorithm. Also, the feature extraction by Haralick texture and local binary pattern (LBP) is recommended. Due to the obtained results, the accuracy of this method is 98.89%, which represents the high potential and efficiency of this method.

## 1. Introduction

The term “cancer” refers to the abnormal growth of some cells. These cells can spread and invade other sections of the human body. The occurrence of this in the breast texture as a mass is called breast cancer. Breast cancer occurs mainly in women, but it may be observed in men, too. After lung cancer, the second cause of death in women is breast cancer. Generally, there are two types of breast cancer based on primary origin: primary tumors and secondary tumors. The primary tumor originates from breast texture cells. In metastatic tumors, the cells become cancerous in another section of the body and spread to the breast through the lymphatic system or bloodstream.

Based on the American Cancer Society (ACS) statistics, the incidence rate for new cases of this disease is 125.3 for females each year (per 100,000 men and women). The death rate value is 20.3 for females per 100,000 men and women each year. The rates are given age-arranged and according to 2012–2016 cases and 2013–2017 death cases [1].

Invasive Ductal Carcinoma (IDC) is the most communal type of this cancer. It begins from cells that put the milk duct into the breast. Then destroys the duct wall and spreads to the nearby breast textures. At this point, it spreads (metastasize) through the lymph system and stream of the blood. Many women with breast cancer diseases are treated by (1) hormone therapy and (2) chemotherapy. Similarly, targeted therapy or radiation is the local treatment. Sometimes, a combination of these treats is used. Early diagnosis of this disease can be very important to cure.

The breast imaging methods commonly used at this time are mammograms, ultrasound, breast MRI, breast tomosynthesis (3D mammography), positron emission tomography (PET), computed tomography (CT) scan, optical imaging tests, electrical impedance imaging (EIT) scans, contrast-enhanced mammography (CEM), and, at last, the chest X-rays. These techniques are used for careful observation of significant things like the shape, size, location, the exact kind of cancer, more details about the stage of cancer or how fast it is growing, and metabolism of breast tumors. Sometimes, a combination of these methods is used for a more accurate diagnosis.

According to recent researches, MRI may locate certain small breast lesions that are occasionally missed by the mammography method. Therefore, it can be a useful diagnostic tool. Nowadays, computer-aided diagnosis (CAD) based on MRI images is used to detect tumors. Hence, this efficient method is more important. Indeed, combining the CAD systems with MRI images is caused to decrease the useless data and aided in fast detection of the tumor. Recently, artificial intelligence based on CAD has been used to improve detection.

Formerly, mammography and MRI image processing for breast tumor diagnosis were based on machine learning techniques and extraction of geometric features. In deep-learning algorithms, the convolutional neural networks (CNNs) are speedily becoming a prevalent technique to process medical images. Deep learning is hierarchical learning and one of the subbranches of machine learning which is for learning high-level data summaries. This emerging method has been noticed more in the artificial intelligence field.

In previous articles, several techniques were presented for breast tumor diagnosis. For instance, Hu et al. [2] proposed a method for feature extractions of (1) (DCE)-MRI and (2) (T2w) sequences to improve breast cancer detection. (DCE)-MRI sequence is the dynamic contrast-enhanced and T2 is the weighted (T2w) MRI sequence for each MR study. Based on the mentioned features, this method was used as a pretrained convolutional neural network (CNN) for classification and final detection between benign and malignant tumors. In conclusion, feature fusion using DCE (*P* value < 0.001) (95% confidence intervals) had statistically better performance.

Ibrahim et al. [3] introduced a segmentation approach for breast tumors in thermal images. They used the chaotic salp swarm algorithm (CSSA) to this. This segmentation algorithm uses the quick-shift technique which clusters the breast thermal image pixels to reach the optimal superpixels. The final results showed that removing the extra parts of the image and keep the breast area. This leads to improving the detection accuracy (92%).

Ibraheem et al. [4] presented a median 2D filter which used to preprocess breast cancer images. Feature extraction was performed by the DWT (discrete wavelet transform) method and then reduced to 13 features. Eventually, a support vector machine (SVM) has been utilized to detect the cancerous mass. Simulation and test results have shown 98.03% accuracy.

Navid et al. [5] recommended a method that uses a threshold-based WCO optimization algorithm. WCO is a metaheuristic algorithm inspired by the FIFA World Cup challenge. Then, the Kapur approach was used to define an objective function. Finally, the candidate solutions were selected from random samples of the search space in the image histogram.

Toğaçar et al. [6] introduced novel deep learning which was developed based on the convolutional neural network. They proposed a model called BreastNet to improve the quality of the classification. The BreastNet was built based on attention modules. The data has been processed by augmentation techniques. The image features are exchanged by various augmentation techniques like shift, rotation, flip change, and brightness. Also, they used the hypercolumn method for the accurate classification of the data. Other sections of the BreastNet pattern model include the pooling, convolutional, dense blocks, and residual. The method obtained 98.80% accuracy.

Alanazi et al. [7] proposed a CNN method that analyzes the hostile ductal carcinoma tissue regions in whole-slide images (WSIs) to automatic detection of the breast cancer. The suggested system using CNN Model 3 obtains 87% accuracy. The five-layer CNN in Model 3 is best suited for this detection. The paper studies the presented technique that applies various convolutional neural network (CNN) architectures to automatically detect breast cancer, comparing the results with those from machine learning (ML) algorithms.

Ma et al. [8] presented that a 1D-CNN model was developed and trained for classification. The Fisher discrimination analysis (FDA) and support vector machine (SVM) classifiers were trained and tested with the same spectral data for comparison. The best classification performance, namely, the overall diagnostic accuracy of 92%, the sensitivity of 98%, and the specificity of 86%, has been achieved by using the 1D-CNN model.  Table 1 indicates some recent studies in breast tumor identification.

**Figure 1:**
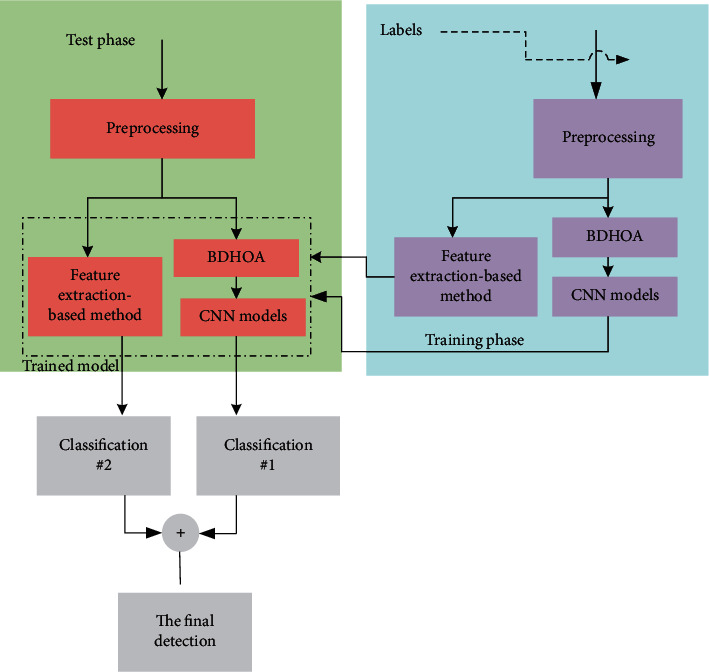
The graphical abstract of the proposed method.

In this study, our main purpose is to provide a twofold system for better diagnosis. The proposed approach includes a CNN model optimized with an improved algorithm, which is performed with a texture feature-based technique as a sequential method. Then, the results are combined to achieve the best result. This computer system reduces the complexity and leads to improving computational performance. Additionally, it solves the problems of the previous literature to achieve the best results [9].  Figure 1 is an overview of the proposed method.

The main contributions of this study are highlighted as follows:

- Optimal comprehensive approach for the automatic detection of breast cancer by the CAD
- Hybrid method to improve the classification performance and efficiency
- A noise reduction and normalization of the data
- CNN classifier amended by Balanced Deer Hunting Optimization Algorithm (BDHOA) for classification
- Haralick texture and local binary pattern for feature extraction
- Independent component analysis for dimension reduction of the features

## 2. Image Preprocessing

First, input breast MRI image data should be simplified and prepared for the next steps. Thus, in the first step, normalization is applied. Hence, the data intensity values are normalized by the min-max method in the scale range 0-1. Here, the size of the image is 250 × 250. Then, the noise reduction method is used to eliminate undesired distortions. Noise reduction is the most important phase of preprocessing. In recent studies, partial equations have been used to reconstruct images. Also, MR images have problems such as electromagnetic (EM) noise emitted from circuits. The main cause of noise in MRI imaging can be of two types: (1) hardware and (2) subject (physiological noise, body motions, cardiac pulsation, respiratory motions, etc.).

To overcome the noise problems of the breast MR images, they must be filtered. Acoustic noise is the main noise in MRI. So, noise removal is important in medical image processing. In this regard, an Intelligent Hybrid Filter is used. This fuzzy-based filter is utilized to eliminate the noise of images. This filter is used in particular for the preprocessing of medical images [8]. The procedure of this filter is summarized as follows [7]:

1. The noisy image is passed in parallel from four noise removal filters
2. X is the input image, and *X*_0_, *X*_1_, *X*_2_, and *X*_3_ are the output of the filters
3. The output of the filters enters the fuzzy-neural system as input
4. Finally, *Y* is the output of the fuzzy-neural system and is the final improved image

## 3. Convolutional Neural Networks

CNN is an abbreviation form of convolutional neural networks. It is one of the branches of deep neural networks. Also, it is highly accurate in image processing, classification, and segmentation. The CNNs are mainly used in machine learning for visual or speech analysis and diagnosis.

Convolutional networks were inspired by biological processes of the (connections between neurons) cat's visual cortex. It is a significant approach in deep learning, wherein several layers are trained purposefully and powerfully [10]. It is more efficient because of its accuracy and fast operation. In computer vision, CNN is one of the most important methods.

Generally, all models of CNN contain three key parts: (1) convolution layer, (2) pooling layer, and (3) fully connected layer, where each layer has a definite task. Also, CNN has two training steps. At first, the input image has been injected into the CNN with a simple dot multiplication between the input and the neuron parameters, which is followed by a convolutional multiplication during each layer. The network error is computed from the output to network training. For this purpose, it compares the network output by using a loss error function and correct solution and computing the error rate. Then, the phase of backpropagation starts based on the amount of calculated error rate, where the derivative of each parameter is obtained using the chain rule.

Also, these components change based on their effect on the network error [9]. After updating the parameters, the next stage is feedforward. These steps are repeated several times in sufficient numbers until the network training is completed. The learning is used to get a certain number of kernel matrixes. In this case, gradient descent was utilized for the selection of the optimal network weights. In network, a ReLU (rectified linear unit) function with *f*(*z*) = max(*z*, 0) is utilized to activate neurons. The output scale is intensely reduced by max pooling [10].

The training error is evaluated to adapt the weight of the neuron and obtain the desired output. The backpropagation step minimizes the cross-entropy loss [11].

The loss is with the following formula:

(1) $$\begin{array}{c} \text{CE}=\overset{N}{\underset{j=1}{\sum }}\overset{M}{\underset{i=1}{\sum }}-D_{j\;}^{\left(i\right)}\log\;z_{j}^{\left(i\right)}, \end{array}$$

where *D*_*j*_ defines the achieved output vector for the *m*^*th*^ class such that $d_{j}=\left(0,...,0,\underset{k}{\underset{︸}{1,\ldots ,1}},0,...,0\right)$ explains the desired output vector and *z*_*j*_^(*i*)^ demonstrate the Softmax function which is shown by the following formula:

(2) $$\begin{array}{c} z_{j}^{\left(i\right)}=\frac{e^{f_{j}}}{\sum_{i=1}^{M}e^{f_{i}}}, \end{array}$$

where *M* describes the sample number, and a weight penalty (*ρ*) has been utilized for extending the function by storing the values of the weight large; that is,

(3) $$\begin{array}{c} L=\overset{N}{\underset{j=1}{\sum }}\overset{M}{\underset{i=1}{\sum }}-D_{j}^{\left(i\right)}\log\;z_{j}^{\left(i\right)}\;+\frac{1}{2}\rho \underset{K}{\sum }\underset{L}{\sum }V_{k,l}^{2}, \end{array}$$

where connection weight is indicated by *V*_*k*_, *k* in layer *l* and *L* shows the layer's totality number, and *K* illustrates the layer for *l* connections. Given that the designed layouts of the CNN are based on tests and errors, it also has problems. In recent years, various types of optimal automatic approaches have been presented for extending the network using bioinspired optimization algorithms [12].

## 4. Deer Hunting Optimization Algorithm (DHOA)

One of the steps is optimization, which is the process of obtaining the “best available” values of a problem. Sometimes, conventional classical optimization algorithms are not able to solve problems correctly and quickly [13]. To overcome this issue, there is a new technique called metaheuristics for fast solutions of the problems such as NP-hard (nondeterministic polynomial-time hard). Metaheuristics can be imitated based on different phenomena from the animals hunting behavior to humankind's social behaviors [14]. In some cases, algorithms are also improved to find the best optimum response. For example, the harmony search algorithm, dolphin swarm algorithm, genetic algorithm, symbiotic organism search, and the world-cup optimization algorithm are used to solve the various types of complex problems [15]. Besides, Yin and Navid suggested a modern bioinspired algorithm that is inspired by hunting deer [16].

The deer's features make their hunting process more difficult. An important feature of deer is their vision. Its visual feature is five times stronger than man's vision. The other remarkable feature of the deer is its sense of smell. This sense in deer is sixty times stronger than human's smell sense.

The deer snores loudly and walks heavily when it realizes the danger of this. This reaction can let another deer know. The deer can also detect the extreme frequency of the sounds well. In the following text, the deer hunting system has been described in detail.

### 4.1. Initialization

The metaheuristic deer hunting algorithm starts with the set known as the hunter, which is a group of random populations, which is defined as follows:

(4) $$\begin{array}{c} Z=\left\{y_{1},y_{2},\ldots ,y_{n}\right\},\;1<j\leq n, \end{array}$$

where the number of hunters or types of solutions is indicated by *n*. Also, *Z* refers to the total hunter population.

### 4.2. Initializing the Parameters

The second stage involves quantifying the main components, the angle of the position of the deer and the angle of the wind. Space is considered a circle. Thus, the wind angle is written in the formula of a circle:

(5) $$\begin{array}{c} \theta_{j}=2\pi \alpha , \end{array}$$

where *α* is a random integer within the limitation {0,1} and *j* describes the present iteration. Also, the angle of the deer location is defined as follows:

(6) $$\begin{array}{c} \varphi_{j}=\pi +\beta . \end{array}$$

Here, *β* shows the angle of the wind.

### 4.3. Position Propagation

During the first iteration, it is usually not possible to find the best solution for the algorithm [17]. However, after generating a random integer and evaluating the cost function from it, the best integer is considered as the optimal solution value [18]. Here, we assumed two parameters, including leader position (*z*_*l*_) as the initial best location of the hunter and the successor position (*z*_*s*_) as the succeeding hunter position.

#### 4.3.1. Propagation Based on the Leader's Position

To get the best position using the initial repetition, the entire population tries to reach the best position by updating their location. Hence, the “encircling behavior” is mathematically formulated by the following equation:

(7) $$\begin{array}{c} Z_{j+1}=Z_{L}-k\times S^{w}\times \left|L\times Z_{L}-Z_{j}\right|. \end{array}$$

In this formula, *Z*_*j*_ and *Z*_*j*+1_ indicate the present and later locations, *S*^*w*^ shows the random integer based on wind velocity in the scope [0, 2], and the coefficient vectors are denoted by *L* and *k*, where the formula is written as follows:

(8) $$\begin{array}{c} k=0.25\times \;\log\left(I+\frac{1}{I_{\max}}\right)\gamma , \end{array}$$

(9) $$\begin{array}{c} L=2\times \delta , \end{array}$$

where *I*_max_ illustrates the peak of repetition, and in the range [−1, 1], *γ* is the random component.

**Figure 2:**
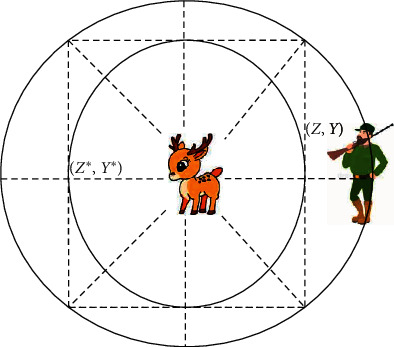
Updating the best position *Z*^*∗*^.

*δ* is the random integer in the range from 0 to 1.  Figure 2 presents the updating position of *Z*^*∗*^, where (*Z*, *Y*) shows the primary location of the hunter that can be updated depending on the prey location.

The updated status will be ongoing to achieve the best situation (*Z*^*∗*^, *Y*^*∗*^) based on the *L* and *K*. The hunters go to the place where the leader is located. If the leader's move was not successful, the hunter stays in his previous position.

The updating of position is according to (9), when *S*^*w*^ < 1. Indeed, hunters can move in all directions regardless of the position angle. Therefore, according to (9) and (10), the hunters can update their locations in each random position.

#### 4.3.2. Propagation Based on the Position Angle

Also, we can expand the space of solving way by considering the location angle. Angle assessment is very important to determine the position of the hunter. So, the successful attack should not be visible to the prey. The visualization of the deer angle (prey) formula is as follows:

(10) $$\begin{array}{c} u_{j}=\frac{1}{8}\times \pi \times \alpha . \end{array}$$

Due to the difference between the angle of the wind and the angle at which the prey is seen, *u* is the parameter that is considered for updating the angle of position:

(11) $$\begin{array}{c} C_{j}=\beta_{j}-u_{j}, \end{array}$$

where *β* illustrates the angle of wind blowing.

Then, to update the position angle parameter,

(12) $$\begin{array}{c} \varphi_{j+1}=\varphi_{j}+C_{j}. \end{array}$$

After obtaining the angle of location, the new location can be calculated using the following formula:

(13) $$\begin{array}{c} Z_{j+1}=Z_{j}-S^{w}\times \left|\cos\left(\varphi_{j+1}\right)\times Z_{l}-Z_{i}\right|. \end{array}$$

The prey does not see the hunter because of the view angle.

### 4.4. Propagation Based on the Position of the Successor

To use the exploration, it is possible to adjust *L* in the behavior of encircling. According to the random first search, the integer of vector *L* cannot be taken into account as more than 1. Thus, the successor location is used for providing a new update of the best solution. An exploration updating formula is given as follows:

(14) $$\begin{array}{c} Z_{j+1}=Z_{s}-k\times S^{w}\times \left|L\times Z_{L}-Z_{j}\right|, \end{array}$$

where *Z*_*s* explains the successor location of hunters at any moment. In this algorithm, the location of the hunters is updated by the best solution in each repetition. The best solution is obtained while |*L*| ≥1. If |*L*| <1, one of the hunters is randomly selected. This method creates an *L* switch, which can modify the mode of the algorithm between the exploitation and exploration stages.

Stuck into the local optimum is a shortage of the original DHO algorithm [19]. In the following, a new modification has been proposed to recover this problem.

### 4.5. The Balanced DHO Algorithm

Here, Lévy flight (LF) is used to evolve the DHO algorithm. Lévy flight is a method that solves the problem of component convergence defect. Lévy flight creates a random walking system that helps to control the local search correctly. The formula is given in the following:

(15) $$\begin{array}{c} Le\left(D\right)\approx D^{-1-\mu },\\ D=\frac{R}{{\left|T\right|}^{1/\mu }},\\ \sigma^{2}={\left\{\frac{\Gamma \left(1+\mu \right)}{\mu \Gamma \left(\left(1+\mu \right)/2\right)}\frac{\sin\left(\pi \mu /2\right)}{2^{\left(1+\mu \right)/2}}\right\}}^{2/\mu }, \end{array}$$

where 0 < *δ* ≤ 2,  *R* ~ *N*(0, *σ*^2^) and *T* ~ *N*(0, *σ*^2^),  Γ(.) presented the gamma, *D* describes the size of the step, the index for Lévy is denoted by *μ*, and *R*/*T* ∼ *N*(0, *σ* 2) describes that Gaussian distribution is used for generating the samples, where the mean value and the variance value are zero and *σ*^2, respectively. Here, *μ*=3/2.

According to the Lévy flight system, the following equation is the new enhanced hunter location:

(16) $$\begin{array}{c} Z_{j+1}^{l}=Z_{j+1}+\left(Z^{*}-RG\right)\times Le\left(\rho \right), \end{array}$$

where *Z*_*j*+1_^*l*^ shows the new location for the agent of search *Z*_*j*+1_ and

(17) $$\begin{array}{c} R=u\left(2\times r-1\right),\\ G=EZ^{\prime }\left(t\right)-Z\left(t\right), \end{array}$$

where *u* is limited within [0, 2] and *r* indicates a random integer at the range from 0 to 1. *Z*′(*t*) represents a random location vector selected for the current population.

For providing guaranteed best solution candidates, fitter agents are kept:

(18) $$\begin{array}{c} {\overset{⟶}{G}}_{el}=\left\{\begin{array}{cc} Z_{j+1}^{l} & F\left(Z_{j+1}^{l}\right)>F\left(Z_{j+1}\right)\\ Z_{j+1} & \text{if not} \end{array}\right., \end{array}$$

**Figure 3:**
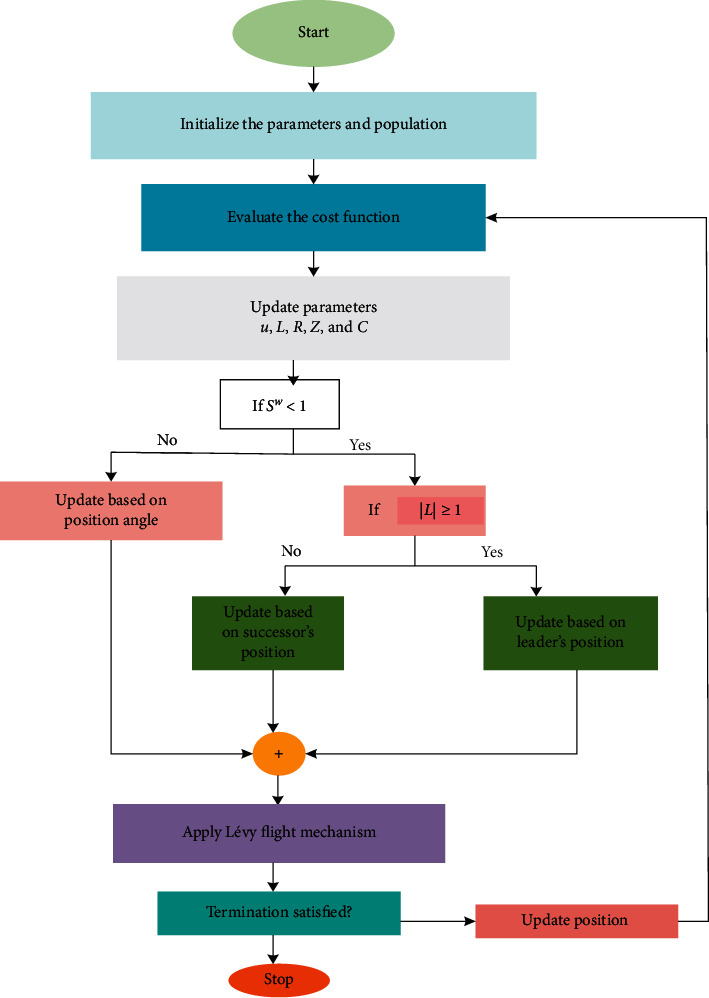
The flowchart diagram of the improved BDHO algorithm.

The following diagram shows the balanced DHOA (BDHOA), which illustrates the steps of this process is given in Figure 3.

## 5. Validation of the BDHO Algorithm

Here, four benchmarks are proposed to analyze the (BDHO) algorithm. Also, several metaheuristic algorithms have been compared with BDHOA. To do this, the benchmarks have been validated on the balanced DHOA, ant colony optimization algorithm (ACO) [20], gray wolf optimization algorithm (GWO) [21], and grasshopper optimization algorithm (GOA) [22, 23], and particle swarm optimization (PSO) [24]. The original DHOA is also given in the table to show the capabilities of BDHOA.

We have simulated by Matlab R2016b with a laptop configuration of 2.20 GHz CPU and 6.00 GB RAM. In this section, the first benchmark function is Rastrigin. Its constraint is [−512, 512] and has the dimension of (30–50) that can be mathematically formulated as follows:

(19) $$\begin{array}{c} F_{1}\left(z\right)=10\times G+\overset{G}{\underset{j=1}{\sum }}\left(z_{j}^{2}-10\times \;\cos\left(2\pi z_{j}\right)\right). \end{array}$$

The second benchmark function is Rosenbrock, which is within [−2.045, 2.045] and has a dimension of 30 to 50. This benchmark can also be formulated as follows:

(20) $$\begin{array}{c} F_{2}\left(z\right)=\overset{G-1}{\underset{j=1}{\sum }}\left(100\left(z_{j}^{2}-z_{j+1}\right)+{\left(z_{j}-1\right)}^{2}\right). \end{array}$$

The third benchmark function with a dimension of 30–50 is within [−10, 10], which is called Ackley. The following formula is related to this benchmark:

(21) $$\begin{array}{c} F_{3}\left(z\right)=20+e-20\times \;\exp\left(-0.2\sqrt{\frac{1}{G}\overset{G}{\underset{j=1}{\sum }}z_{j}^{2}}\right)-\exp\left(\frac{1}{G}\overset{G}{\underset{j=1}{\sum }}\cos\left(2\pi z_{j}\right)\right). \end{array}$$

Sphere benchmark function is the fourth which has [−512, 512] constraint and 30–50 dimensions.

The formula is shown in the following:

(22) $$\begin{array}{c} F_{4}\left(z\right)=\overset{G}{\underset{j=1}{\sum }}z_{j}^{2}. \end{array}$$

The comparison result, according to (1) mean deviation (MD) and (2) standard deviation (SD), has been demonstrated in Table 2.

The table above shows that the mean deviation and standard deviation in the BDHOA method are less and this result is convenient. Also, it can be observed that BDHOA gives the best results compared with the original DHOA. Due to that, it can be useful for obtaining an optimum solution.

### 5.1. Breast Tumor Classification Based on the Proposed Method

The CNN training is mostly performed according to the backpropagation. To overcome the issue of stuck in the local optimum, several methods are presented. In this section, to reduce the network error, the proposed BDHO algorithm is employed instead of the backpropagation approach. The purpose of using this metaheuristic algorithm for CNN is to minimize the value of the mean square error (MSE) function. The formula of the MSE function is as follows:

(23) $$\begin{array}{c} \text{MSE}=\frac{1}{T}\overset{M}{\underset{j=1}{\sum }}\overset{N}{\underset{i=1}{\sum }}{\left(a_{j}^{i}-b_{j}^{i}\right)}^{2}, \end{array}$$

where *a*_*j*_^*i*^ represents the *j*^*th*^ for network output and *b*_*j*_^*i*^ illustrates the *j*^*th*^ of the desired integer of the CNN during the *t* period. *N* signifies the value for layers of the output and *M* indicates the data number in the formula. CNN technique can be very useful in the rapid detection of breast tumors in MR images. In this study, CNN classification is used with two models of CNN and different classifiers [5]. This classification includes (1) extraction of the features and (2) features dimension decrease. This is briefly exhibited in the following.

## 6. Extracting Features

In image processing, feature extraction means converting image data into usable information for the next stages [25]. This is performed by extracting some general or particular features of the input image [26]. Among some types of feature extraction techniques, the texture technique gives more information with details on the spatial arrangement and intensities of colors. Also, this method has some fans in medical imaging. To do feature extraction in this research, two features are used: (1) Haralick features and (2) local binary pattern (LBP). In the following, these two methods are briefly explained.

### 6.1. Local Binary Pattern Features

The operator of LBP selects binary integers of the pixels, then compares these values with their neighbor pixels and decimal numbers, and finally, encodes the surrounding local structure of each pixel.

In the binary labeling step, the resultant strictly negative have been encoded with 0 and the other values encoded with 1. The achieved binary numbers (codes) from the LBP feature are in the clockwise direction. The final extracted binary values are assumed as local binary patterns codes. The final values extracted are binary and called local binary patterns or LBP codes.

### 6.2. Haralick Texture Features

The Haralick feature is a statistical feature that is evaluated from the gray level cooccurrence matrices (GLCM). The purpose is to evaluate the matrix and computes the neighboring gray level cooccurrence in the input image. The GLCM explains the information about a square matrix in the region of interest (ROI) that illustrates a correlation among the reference pixel with a presented intensity integer and the pixels around it that are located in various directions. In this study, four directions, in 0°, 45°, 90°, and 135°, have been employed for the pixels, and the average integers have been used as last the Haralick features.

### 6.3. Dimension Reduction of the Features Based on ICA

**Figure 4:**
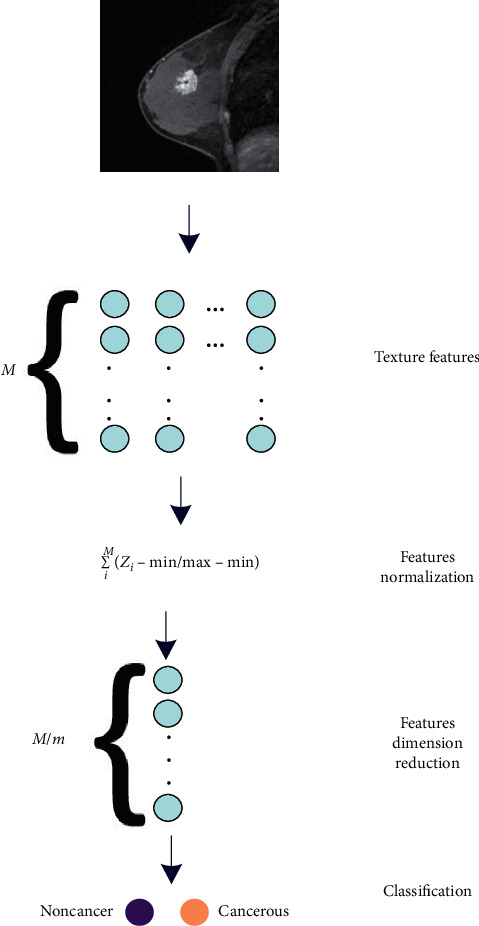
A schematic of the feature extraction-based method.

At this stage, given that the data volume in the feature is high, to achieve the desired volume, the data reduction method is used which also leads to simplification. To do this, feature dimensions are reduced by the independent component analysis (ICA) method. The ICA is a computational methodology for tight-fitting of the concealed factors, which underlie a series of signals. ICA introduces a reproductive model for the large database of MRI. By considering the ICA property, which is a method to separate the blind source, and assuming that the subfactors are non-Gaussian signals and also these subcomponents are free (independent) from each other, the ICA is a very powerful algorithm for analyzing and evaluating principal parameters. The difference is that ICA's ability is to find the underlying sources, even if the classic methods lead to failing. In this algorithm, measurements are given as an array of time series. The phrase Blind Source Separation (BSS) is employed for characterizing the breast waves recorded by several sensors. Finally, the input of the classifier is the data image, which is divided into two sections: training and testing images. After injecting into the classifier, the classifier trains them and predicts the label for test images.  Figure 4 indicates a diagram of the feature extraction-based method.

### 6.4. Final Simulations

The final step is to classify the obtained results from the proposed feature-based techniques and the CNN model. The main goal is to propose an accurate and efficient method to detect breast tumors from MR images. The approach of classification is briefly described in the following.

At the first step, the diagnosis results of hybrid technique (proposed CNN and feature extraction-based) have been collected. After that, the results of the suggested CNN are checked out. If the output is labeled as a tumor, the output will be exhibited as cancer. Otherwise, if the output of the presented CNN is labeled as healthy, the features of the MR image are checked out again according to the feature extraction-based method. In this condition, if the output image has been diagnosed as a tumor, the output will be labeled as cancer, or else, it will be diagnosed as healthy.

## 7. Results and Discussion

### 7.1. Database Description

This method aims to quickly detect the breast tumors in MRI by using MATLAB R2016b software with a system configuration of 2.20 GHz CPU and 6.00 GB RAM. The main idea is to design an optimized CNN (convolutional neural network) to achieve promising results. To validate this technique, it is implemented on the DCE-MRI dataset, which is usually used for analyses of classification efficiency. The DCE-MRI dataset includes a set of 219 breast MR images that is collected from 105 different patients with breast cancer (angiosarcoma, inflammatory, DCIS, ILC, and LCIS) (55 tumor-like and 50 non-tumor-like malignant lesions), and 114 DCE-MRI were detected to be normal. In MATLAB, image size is 512 × 512 pixels. The presented scheme (optimized CNN) and feature extraction-based method are designed for analyzing the MR images.

### 7.2. Simulation Results

There are several types of performance analysis to evaluate classification. One of these analyzes is accuracy. The accuracy determines the proportion of the correctly classified image number to the total image number. The results of the analysis of accuracy for the studied methods, including the feature-based method, optimized CNN, and the hybrid method (feature-based and optimized CNN), are indicated in Table 3.

As can be observed from Table 3, the data presents that the highest efficiency has been achieved when hybrid feature-based/optimized CNN has been utilized for classification. The accuracy of classification can be considered as an efficient indicator to determine the performance of the method when the test dataset contains equal numbers of samples from the classes. The results indicate the efficiency of the proposed system in the rapid diagnosis and timely treatment of the patient.

To get more evaluations, confusion matrices have been used for performance analysis of the breast tumor classification. A confusion matrix is a table with two dimensions which is usually employed for determining the classification efficiency and performance, on a test set to define the true values.  Table 4 illustrates a sample confusion matrix for hybrid feature-based/optimized CNN. This table is based on an investigation on breast cancer: angiosarcoma, inflammatory, and ductal carcinoma in situ (DCIS).

Several indicators have been used for determining the efficiency of the classifier, particularly for each cancer tumor class [27]. The critical indicators in the classification report are specificity, precision, and sensitivity which are obtained from the following formulas:

(24) $$\begin{array}{c} \text{precision}=\frac{\text{TP}}{\text{TP}+\text{FP}},\\ \text{sensitivity}=\frac{\text{TP}}{\text{TP}+\text{FN}},\\ \text{specificity}=\frac{\text{TN}}{\text{TP}+\text{FP}}, \end{array}$$

where the following parameters show some classified cases: FP, false positives; TP, true positives; FN false negatives; and TN, true negatives.

Table 5 shows the final results of using the proposed technique once the optimized and the feature-based method classifier are together for the detection goal.

In Table 5, the integer of the specificity for all the datasets is high, which illustrates the correct identifying samples without the specific disease. The proposed method is also compared with two types of well-known methods.  Table 6 provides a comprehensive comparison, according to some different state of the art for classification techniques [28]. In Table 6, it is clear that the precision parameter in the proposed method is better and higher than other methods.

Briefly, it is observed that once using the suggested method, the value of system efficiency indicators (precision, sensitivity, and specificity) is increased.

## 8. Conclusions

A new comprehensive approach was proposed for the automatic detection of breast tumors. The method is a hybrid model, including an optimized design of a convolutional neural network and feature extraction-based technique to improve the classification efficiency. In this study, preprocessing steps are applied, which eliminate noise and simplifies classification. Additionally, it leads to an increase in the quality of the dataset. Thus, the value is also normalized. Also, the feature extraction-based method was based on Haralick texture features. This method was used with independent component analysis (ICA) to reduce the dimension of the features. Simulations were performed according to the DCE-MRI dataset. The results were compared by various states of the method. Also, other methods were compared to indicate the system's efficiency. It is also possible to increase the accuracy of the study by using a variety of other metaheuristic algorithms. Furthermore, deep convolutional neural network model can be used for further research, to classify the breast cancer images. The final satisfactory results stated the advantage of the suggested approach toward the other methods. In the future, we will examine the proposed technique on a different dataset. The proposed method can be generalized to the design of high-performance computer-aided diagnosis systems for other medical imaging tasks in the future.

## Figures and Tables

**Table 1:** Recent studies in breast tumor identification.

Authors	Year	Approach	Dataset	Average accuracy (%)
Ibraheem et al. [4]	2019	Feature extracted with DWT and then reduced to 13 features	Reference image database to evaluate response (RIDER)	98.03
		Classification: CAD-SVM		
Navid et al. [5]	2020	Segmentation WCO based on image thresholding method	MIAS	87.5
Toğaçar et al. [6]	2020	Classification: hypercolumn technique	BreakHis	98.80
Ibrahim et al. [3]	2020	Segmentation based on a chaotic Salp swarm algorithm (CSSA)	Mastology research with Infrared image (DMR-IR)	92
Hu et al. [2]	2020	Feature: DCE and T2w sequences	mpMRI	95
		Classification: CNN- SVM		
Alanazi et al. [7]	2021	Classification: CNN- SVM	Kaggle 162 H&E	87
Ma et al. [8]	2021	Classification: 1D-CNN model	Spectral data	98

**Table 2:** Analysis of compared methods by dimensions of 30.

Benchmark		BDHOA	DHOA	ACO [14]	GWO [15]	Goa [16]	PSO [24]
*f*_1_	MD	0.00	3.20	67.19	69.80	1.58	4.58
	SD	0.00	3.6	1.50	9.12	3.14	1.13

*f*_2_	MD	6.53	9.58	40.12	89.3	15.22	12.43
	SD	1.88	2.26	24.10	46.15	5.47	2.71

*f*_3_	MD	0.00	5.23*e* − 16	4.11*e* − 3	9.47	2.92*e* − 4	7.12*e* − 9
	SD	0.00	2.8*e* − 6	1.58*e* − 2	3.10	1.5*e* − 3	0.00

*f*_4_	MD	0.00	1.32*e* − 9	1.25*e* − 4	5.29*e* − 5	7.26*e* − 9	8.97*e* − 12
	SD	0.00	15.30*e* − 18	3.65*e* − 5	4.89*e* − 5	2.73*e* − 9	10.14*e* − 15

**Table 3:** Results of the accuracy according to the methods in breast tumor diagnosis on the DCE-MRI database.

Method	Accuracy	Variance (%)
Feature-based	93.15	0.6
Optimized CNN	98.65	0.5
Hybrid feature-based/optimized CNN	98.89	0.4

**Table 4:** Confusion matrix of the hybrid feature-based and optimized CNN structure.

		Predicted		
		Angiosarcoma	Inflammatory	DCIS
Actual	Angiosarcoma	721	12	10
	Inflammatory	27	1393	3
	DCIS	3	0	896

**Table 5:** Results of the proposed technique for detection.

Tumor type	Precision	Sensitivity	Specificity
Angiosarcoma	95.16	95.79	97.42
Inflammatory	97.00	97.07	98.55
DCIS	98.25	98.30	99.15

**Table 6:** The comparison of precision for comparative approaches on the DCE-MRI dataset.

	Angiosarcoma	Inflammatory	DCIS
Mahmuda Rahman	93.06	90.98	96.50
Amira Mofreh Ibraheem	92.70	97.09	92.84
Proposed method	93.99	98.00	96.95

## Data Availability

The dataset images can be found at https://wiki.cancerimagingarchive.net/display/Public/QIN+Breast+DCE-MRI.
